# Evaluation of the Anti-Inflammatory Effect of Chalcone and Chalcone Analogues in a Zebrafish Model

**DOI:** 10.3390/molecules18022052

**Published:** 2013-02-05

**Authors:** Yau-Hung Chen, Wei-Hua Wang, Yun-Hsin Wang, Zi-Yu Lin, Chi-Chung Wen, Ching-Yuh Chern

**Affiliations:** 1 Department of Chemistry, Tamkang University, 151, Yingzhuan Road, Danshui Dist., New Taipei City 25137, Taiwan; E-Mails: faoxmax@hotmail.com (W.H.W.); shes060215@yahoo.com.tw (Z.Y.L.); 2 Division of Basic Research, Koo Foundation Sun Yat-Sen Cancer Center, Taipei 11259, Taiwan; E-Mail: yunhsin_wkimo@yahoo.com.tw; 3 Department of Mathematics, Tamkang University, Danshui Dist., New Taipei City 25137, Taiwan; E-Mail: ccwen@mail.tku.edu.tw; 4 Department of Applied Chemistry, National Chia-Yi University, Chia-Yi 60004, Taiwan

**Keywords:** anti-inflammation, chalcone, fin, zebrafish

## Abstract

The aim of this study was to investigate novel chalcones with potent anti-inflammatory activities *in vivo*. Chalcone and two chalcone analogues (compound **5** and **9**) were evaluated using a caudal fin-wounded transgenic zebrafish line “Tg(*mpx*:*gfp*)” to visualize the effect of neutrophil recruitment dynamically. Results showed that treatment with compound **9** not only affected wound-induced neutrophil recruitment, but also affected Mpx enzymatic activity. Moreover, protein expression levels of pro-inflammatory factors (Mpx, NFκB, and TNFα) were also regulated by compound **9**. Taken together, our results provide *in vivo* evidence of the anti-inflammatory effects of synthesized chalcone analogues on wound-induced inflammation.

## 1. Introduction

Chalcone (1,3-diphenyl-2-propen-1-one), is a phenolic compound abundant in vegetables. Natural occurring chalcones as well as synthetic chalcone analogues have demonstrated many pharmaceutical effects, including anti-inflammatory, anti-oxidant, anti-parasite, and anti-tumor activities [1,2,3,4,5,6,7]. Recently studies revealed that chalcones (chalcone and synthetic chalcone analogues) can inhibit NO synthesis and inducible NO synthetase (iNOS) and cycloxygenase 2 (COX-2) protein expression in lipopolysaccharide (LPS)-stimulated cells, and indicated the importance of chalcones as anti-inflammatory agents [8]. However, current knowledge regarding the anti-inflammatory effects of chalcones in vertebrates has all been reported *in vitro.* Thus, it is essential to establish an effective animal model to study the *in vivo* the anti-inflammatory effects of chalcones.

Inflammation is a complex biological event of a tissue response to a harmful stimulus (bacterial infection, burn or wound, for example). Acute inflammation usually involves dynamic regulation of pro-inflammatory mediators (Mpx, NFκB, TNFα) and the recruitment of white blood cells to harmed sites [9]. Neutrophils are one type of white blood cells which can migrate towards the harmed sites and are considered as the hallmark of acute inflammation [9]. For this reason, monitoring the number and the migration activity of neutrophils is an efficient way to evaluate acute inflammatory responses.

The optical transparency of zebrafish embryos allows noninvasive and dynamic imaging of the inflammation process *in vivo.* Especially, a transgenic zebrafish line Tg (*mpx*:*gfp*) expressing green fluorescent protein (GFP) under the control of neutrophil-specific *mpx* promoter enables us to count the number and to monitor the migration activity of neutrophils more efficiently [10]. In this study, a wounded zebrafish model was used to assess the anti-inflammatory effects of chalcones (chalcone and chalcone analogues) on wound-induced inflammation *in vivo*. We also evaluated the Mpx expression by histochemical staining, and examined the protein levels of three evolutionarily conserved pro-inflammatory factors (Mpx, NFκB, and TNFα) upon chalcones treatment.

## 2. Results and Discussion

### 2.1. Chemistry

**Figure 1:**
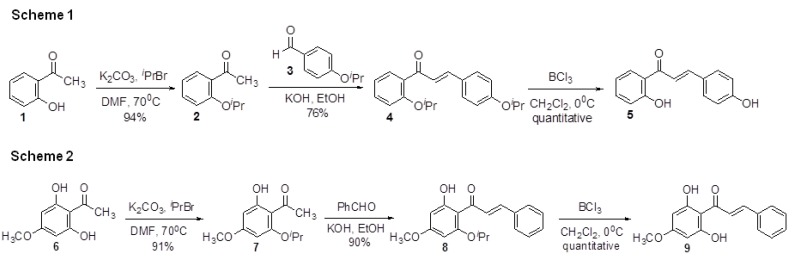
Synthesis of compound **5** and compound **9**.

For this study, we have developed simple methods for the synthesis of chalcone derivatives **5** and **9** from *O*-isoproxyacetophenones **2** and **7**. These two compounds are known compounds [11,12]. The aldol intermediates were obtained by using the similar procedure described previously [13]. Unfortunately, the reaction yield using this method was very low (~15%). Yields didn’t improve on using different bases (KOH/NaOH). It is possible that intramolecular hydrogen bonding such as that observed in **1** or **6** prevents the aldol reaction. Therefore, *O*-isoproxyacetophenones **2** and **7** were used as the starting material for our synthesis. The preparation of *O*-isoproxyacetophenones **2** and **7** was straightforward. Acetophenones **1** and **6** were protected with isopropyl bromide and potassium carbonate in DMF in excellent yield. (Figure 1, Schemes 1 and 2) The isolated product **2** and **7** were then reacted with appropriate benzaldehydes with 5N KOH whereupon intermediates **4** and **8** were isolated in 76% and 90% yields. The *O*-isopropyl ether was removed quantitatively with BCl_3_ to afford the target chalcones **5** and **9**. Inspection of the ^1^H-NMR spectra of chalcones clearly indicated that they were *trans* configured (*J*_H__α,H_ = 15–16 Hz).

### 2.2. Effects of Chalcones on Wound-Induced Neutrophil Recruitment and Mpx Enzymatic Activity

**Figure 2:**
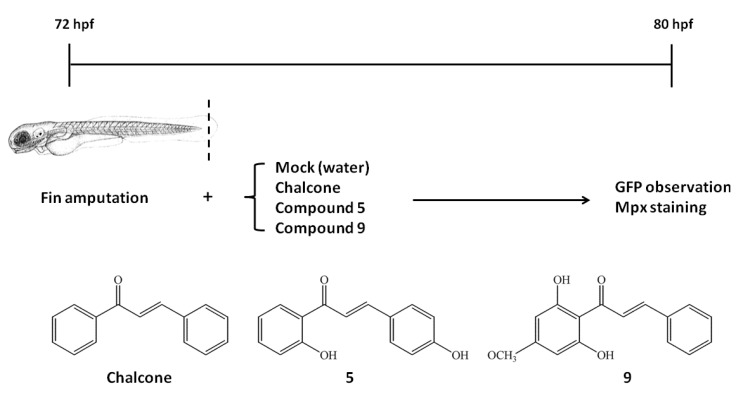
Schematic representation of experimental protocols performed in this study. Fins of Tg(*mpx:gfp*) zebrafish embryos were amputated by 72 hpf, and were cultivated with (chalcone, compound **5**, and **9**) or without chemicals (mock control) for 8 h. By 80 hpf, embryos were collected for fluorescent recording, Mpx staining or western blotting experiments. Structures of chalcone, compound **5** and compound **9** were listed in the bottom.

We have previously developed a protocol to detect the level of wound-induced neutrophil recruitment in transgenic zebrafish Tg (*mpx*:*gfp*) embryos [14]. The same protocol was employed to evaluate the newly synthesized chalcone analogoes **5**, and **9** with chalcone for comparison (Figure 2).

**Figure 3:**
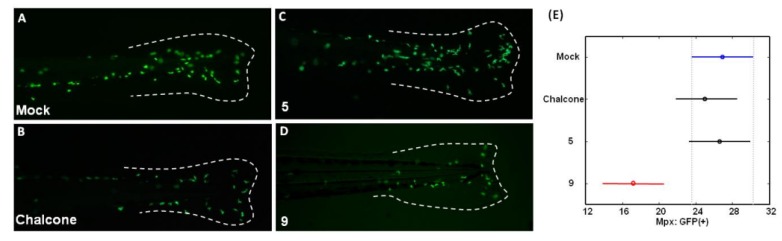
Myeloperoxidase (Mpx) expression in zebrafish larvae in response to wounding or chalcones (chalcone, compound **5** and **9**) treatment. (**A**–**D**) The distribution of neutrophils in living Tg(*mpx:gfp*) zebrafish larvae using GFP fluorescence as a marker. Dashed line outlines the region of caudal fin. (**E**) The Tukey-Kramer HSD reports the mean numbers of Mpx and their 95% confidence intervals for four treatment groups (‘Mock’, ‘chalcone’, ‘**5**’, ‘**9**’).

As shown in Figure 3, green fluorescent neutrophils (hereafter referred to as Mpx:GFP(+) cells) tended to migrate toward the wound site upon fin amputation in wounded zebrafish (Figure 3A, Mock). Upon treatment with 1 ppm of chalcone and compound **5** for 8 h, no obvious increase in Mpx:GFP(+) cells was observed (Figure 3B,C) compared with the wounded mock control group (Figure 3A). In contrast, the difference between the compound **9**-treated group (Figure 3D) and wounded mock control control (Figure 3A) was reduced. Statically, the ANOVA method was first applied to examine the effect of treatment (‘Mock’, ‘chalcone’, ‘**5**’, ‘**9**’; n = 15) on the mean number of Mpx. Of primary interest here is a test of the hypothesis of no difference in mean numbers of Mpx between treatment groups. The test reports a *p-*value of 0.0007, indicating a highly significant difference between treatment groups. To pinpoint which treatment means are significantly different from each other, the Tukey-Kramer HSD test was further used for pairwise comparisons. Figure 3E presents the mean numbers of Mpx and their 95% confidence intervals for four treatment groups. It reports the mean numbers of Mpx for ‘Mock’, ‘chalcone’, ‘**5**’, ‘**9**’ groups are 26.93, 25.00, 26.64, and 17.21 with common stand error being 1.78, and also identified the mean number of Mpx for ‘Mock’ group differs significantly from the ‘**9**’ group at familywise error rate 0.05.

**Figure 4:**
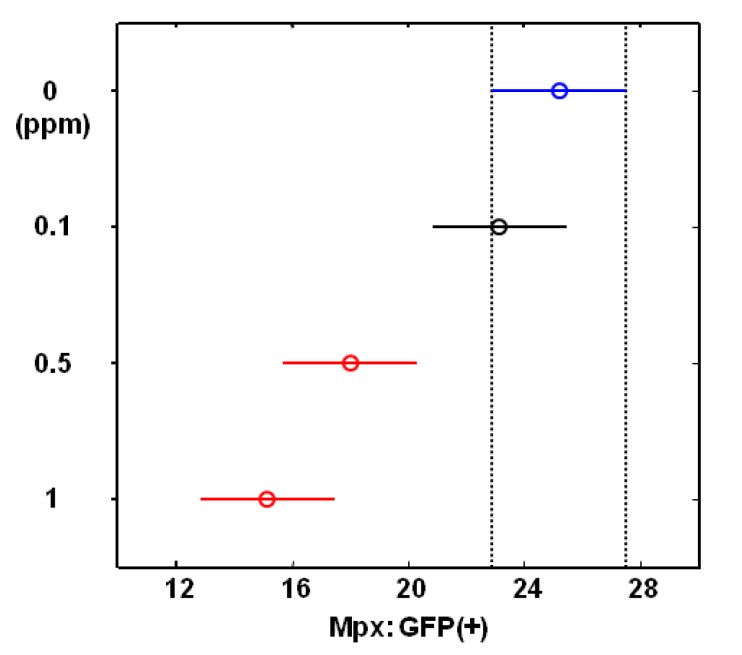
The Tukey-Kramer HSD test reports the mean numbers of Mpx:GFP (+) and their 95% confidence intervals for each group which were treated with different doses of compound 9 (0, 0.1, 0.05 and 1 ppm).

We further treated wounded zebrafish with different doses of compound **9**, followed by statistical analysis. The ANOVA method for examining the effect of compound **9** dose (‘0’, ‘0.1’, ‘0.5’ and ‘1’ ppm) on the mean number of Mpx reported a highly significant difference between compound **9** dosage groups (p-value < 0.0001). The Tukey-Kramer HSD test for pairwise comparisons of dosage groups reported the mean numbers of Mpx (standard error, sample size) for four dose groups (‘0’, ‘0.1’, ‘0.5’ and ‘1’) are 25.21 (1.22, n = 14), 23.14 (1.22, n = 14), 18.00 (1.22, n = 14), and 15.14 (1.22, n = 14), respectively. Figure 4 presents the mean number of Mpx and their 95% confidence interval for these dosage groups and concludes that the mean number of Mpx for ‘0.5’ and ‘1’ groups differ significantly from the ‘0’ group at familywise error rate 0.05. These *in vivo* results demonstrated that wound-induced neutrophil recruitment in living zebrafish was attenuated by chalcone analogue (compound **9**) treatment in a dose-dependent manner.

**Figure 5:**
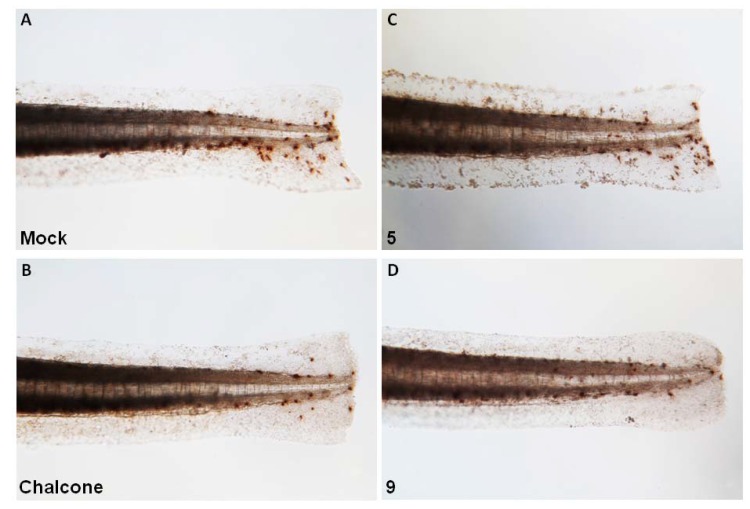
Endogenous myeloperoxidase (Mpx) activity in zebrafish larvae in response to wounding or chalcones (chalcone, compound **5** and **9**) treatment. Fin-amputated zebrafish embryos derived from the mock control (**A**) or the chemicals-treated groups (**B**–**D**) were stained with benzidine dihydrochloride to visualize endogenous Mpx activities.

To further evaluate the effect of chalcone (chalcone, **5** and **9**) treatment on Mpx enzymatic activity in neutrophils, endogenous Mpx enzymatic activity in the zebrafish embryos derived from each experimental group was observed by histochemical staining with peroxidase substrate benzidine. Positively-stained cells [hereafter indicated as Mpx(+) cells] revealed endogenous Mpx enzymatic activities *in vivo* and *in situ*. As shown in Figure 5A, an increased cell number of Mpx(+) was observed in wounded zebrafish (Figure 5A, Mock), revealed an increase in Mpx enzymatic activity. Upon compound **5** treatment, no evident decrease of the Mpx(+) cells was observed in compound **5**-treated group (Figure 5B) versus mock control (Figure 5A). However, a decrease of the Mpx(+) cells was observed both in chalcone and compound **9**-treated group (Figure 5C,D, *vs.* 5A). Taken together, we suggest that: (1) chalcone is able to attenuate endogenous Mpx enzymatic activity rather than the wound-induced neutrophil recruitment *in vivo*; and (2), compound **9** is able to suppress Mpx enzymatic activity as well as neutrophil recruitment.

### 2.3. Molecular Mechanism of Anti-inflammatory Effects of Compound ***9***

**Figure 6:**
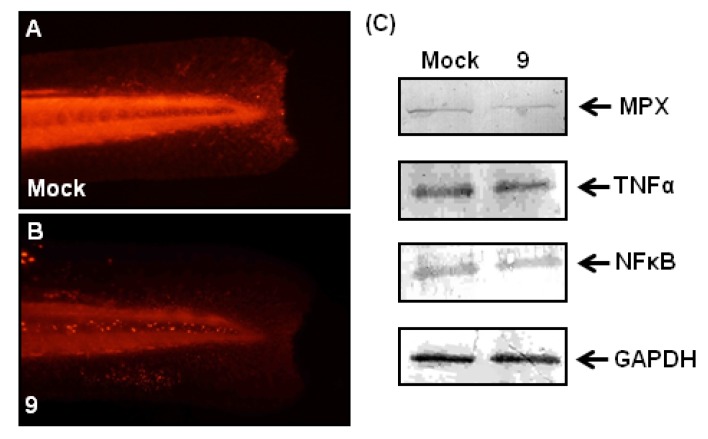
Effects of compound **9** on fin-amputated zebrafish larvae. Fin-amputated zebrafish embryos derived from the mock control (**A**) or the compound **9**-treated groups (**B**) were stained with antibody against Mpx. (**C**) Results of western blot analysis of the mock control and the compound **9**-treated embryonic lysates using antibodies against Mpx, NFκB, TNFα and GAPDH.

To further investigate the molecular mechanism of anti-inflammatory effects of compound **9**, we examined the effects of compound **9** on Mpx, NFκB and TNFα at the protein level (Figure 6). The protein expression level of Mpx in zebrafish larvae at 80-hpf (8 hours post-treatment) were detected by whole-mount immunostaining using antibody against Mpx (Figure 6A,B). After fin amputation followed by compound **9** treatment, Mpx expression appeared to decrease compared with mock control (no-treatment) group (Figure 6A *vs.* 6B). These immunostaining results revealed that compound **9** slightly attenuated the protein expression of Mpx in wound-induced inflammation, which is consistent with the above-mentioned Mpx enzymatic activity revealed by histochemical staining. On the other hand, western blotting results revealed that the protein expression amount of NFκB and TNFα change little, but amount of Mpx is reduced (Figure 6C). Taken together, our results suggested that the anti-inflammatory effects of compound **9** on wound-induced inflammation were mediated through the regulation of Mpx.

## 3. Experimental

### 3.1. General

Proton NMR spectra were recorded at 300 MHz Varian Mercury-300 NMR spectrometer. Carbon NMR spectra were recorded at 75 MHz Varian Mercury-300 NMR spectrometer. Proton and carbon chemical shifts are reported on the delta scale as parts per million (ppm) downfield from tetramethylsilane (TMS) as internal reference. All reagents were used as obtained commercially.

### 3.2. Synthesis of New Chalcone Analogues

Synthesis procedure examples:

*3-(4-Hydroxyphenyl)-1-(2-hydroxyphenyl)propenone* (**5**).^1^H-NMR (CDCl_3_, 300MHz) δ: 13.96 (1H, s, -OH), 7.93 (1H, d, *J =* 8.7 Hz), 7.90 (1H, d, *J =* 15.8 Hz), 7.57 (2H, d, *J =* 8.7 Hz), 7.52 (1H, d, *J =* 15.8 Hz), 7.49 (1H, t, *J =* 8.7 Hz), 7.0–6.8 (4H, m); ^13^C-NMR (CDCl_3_, 75MHz) δ: 193.8, 163.5, 158.3, 145.4, 136.3, 130.8, 129.6, 124.6, 122.2, 118.8, 118.6, 116.1, 115.9

*1-(2,6-Dihydroxy-4-methoxyphenyl)-3-phenylpropenone* (**9**): ^1^H-NMR (d_6_-acetone, 300 MHz) δ: 12.07 (2H, bs, -OH), 8.25 (1H, d, *J =* 15.6Hz), 7.73 (1H, d, *J =* 15.6 Hz), 7.71–7.66 (2H, m), 7.46–7.40 (3H, m), 6.04 (2H, s), 3.81 (3H, s); ^13^C-NMR (d_6_-acetone, 75MHz) δ: 193.4, 167.2, 165.4, 142.9, 136.4, 130.9, 129.8, 129.1, 128.3, 106.2, 94.6, 55.8

### 3.3. Zebrafish Larvae, Fin Amputation and Chalcones Treatment

Zebrafish larvae (wild-type, WT; AB strain) and Tg(*mpx*:*gfp*) were raised at 28.5 °C and staged according to standard protocols [15,16]. Fin amputation was carried out by cutting out half of the caudal fin as shown in Figure 2. Chalcone (C_15_H_12_O, Sigma-Aldrich), compound **5** and **9** were dissolved in dimethylsulfoxide (DMSO) as stock solution (2,500 ppm), and diluted to 1 ppm for treatment. Wounded zebrafish larvae were randomly divided into no-treatment “control” group (Mock), chalcone, compound **5**, and compound **9**-“treated” group (chalcone, **5**, **9**). Each group was exposed to DMSO (no treatment, Mock) or chalcones (chalcone, **5**, **9**) in the dark at 28.5 °C for 8 h (Figure 2).

### 3.4. Myeloperoxidase Staining, Antibody Labeling and Western Blotting

Myeloperoxidase staining and antibody labeling experiments were performed as previously described with minor modifications [14,17,18,19,20,21]. Western blotting followed standard procedures except for the use of antibodies against Mpx (AnaSpec), NFκB (AnaSpec), TNFα (AnaSpec) or GAPDH (Santa Cruz) as primary antibodies.

### 3.5. Statistical Analysis

All analyses in this study were carried out according to Matlab software (version 7.7 R2008b). The ANOVA (analysis of variance) test was applied to examine the effect of treatment (or dosage) on the mean number of Mpx. The p-value reported by ANOVA method associates with the null hypotheses that samples in all treatment (or dosage) groups are drawn from the same population. The Tukey-Kramer HSD (honestly significant difference) test was further used to compare the population marginal mean number of Mpx for each treatment (or dosage) group. A significance level 0.05 was used in all statistic analyses and a familywise error rate 0.05 was controlled for Tukey-Kramer HSD test.

## 4. Conclusions

This zebrafish model of wound-induced inflammation can be readily applied to *in vivo* screening of the therapeutic potential of anti-inflammatory compounds. This study suggested that some chalcones, especially compound **9**, may have the potential to be developed as an anti-inflammatory agent. However, further studies are necessary to establish such as examination of the underlying molecular mechanisms and direct targets at the transcriptional or post-transcriptional level in mammals.
